# The Current SI Seen From the Perspective of the Proposed New SI

**DOI:** 10.6028/jres.116.022

**Published:** 2011-12-11

**Authors:** Barry N. Taylor

**Affiliations:** National Institute of Standards and Technology, Gaithersburg, MD 20899

**Keywords:** BIPM, CCU, CGPM, CIPM, International System of Units, New SI, SI, SI reference set of constants, units

## Abstract

A revised International System of Units (SI) proposed by the International Committee for Weights and Measures is under consideration by the General Conference on Weights and Measures for eventual adoption. Widely recognized as a significant advance for both metrology and science, it is defined via statements that explicitly fix the numerical values of a selected set of seven reference constants when the values of these constants are expressed in certain specified units. At first sight this approach to defining a system of units appears to be quite different from that used to define the current SI. However, by showing how the definitions of the seven base units of the current SI also fix the numerical values of a set of seven reference constants (broadly interpreted) when the values of these constants are expressed in their coherent SI units, and how the definition of the current SI can be recast into the same form as that of the revised SI under consideration, we show that the revision is not as radical a departure from the current SI as it might initially seem.

## 1. Introduction

Although it is assumed that the reader is familiar with the current International System of Units (SI), for convenience, Sec. 4, Appendix A, which is based on Refs. [1, 2], provides a brief review of the current SI and the international bodies responsible for it, namely, the CGPM, CIPM, BIPM, and CCU (General Conference on Weights and Measures, International Committee for Weights and Measures, International Bureau of Weights and Measures, and Consultative Committee on Units, respectively). However, the actual definitions of the current SI base units, second s, meter m, kilogram kg, ampere A, kelvin K, mole mol, and candela cd, are not included in Appendix A, because they are given and used in Secs. 2.1–2.7 of the main text. This order of base units is employed throughout the paper so that no unit definition depends on another unit whose definition appears later in the sequence, rather than the traditional order m, kg, s, A, K, mol, and cd.

### 1.1 The New SI

Over at least the last dozen years there has been extensive discussion in various international and national forums concerning the possible redefinition of the SI in order to meet the challenges of the 21st century [3–9]. The culmination of this discussion is Draft Resolution A considered by the 24th CGPM at its October 2011 meeting [10], which was prepared by the CIPM upon the recommendation of its CCU. Reference [11] reviews and discusses in some detail the development of and the basis for the proposals in Draft Resolution A and summarizes the many benefits of the “New SI” as it has come to be called. Indeed, a broad consensus now exists in both the international metrological and scientific communities in support of the adoption of the New SI [12]. Section 5, Appendix B gives its definition as presently envisioned, the formal adoption of which could be at the 25th meeting of the CGPM in 2014.

As can be seen from Appendix B, the New SI is based on the set of seven reference constants Δ*ν* (^133^Cs)_hfs_, the ground state hyperfine splitting frequency of the cesium 133 atom; *c*, the speed of light in vacuum; *h*, the Planck constant; *e*, the elementary charge (charge of a proton); *k*, the Boltzmann constant; *N*_A_, the Avogadro constant; and *K*_cd_, the luminous efficacy of monochromatic radiation of frequency 540 × 10^12^ Hz. The principal purpose of this paper is to show fully and clearly that the current SI can also be understood as being based on a set of seven reference constants and that its current definition can be recast, as demonstrated in Sec. 6, Appendix C, into the same form of wording as the New SI as given in Appendix B. It is thus shown that the New SI, although widely recognized as a major advance, is not the radical departure from the current SI as it might first appear. (Appendices are used to keep the main text concise and focused on the paper’s principal purpose.)

### 1.2 Quantity, Value of a Quantity, and Numerical Value of a Quantity

As a prelude, we briefly recall the relationship between a quantity, its value, and its numerical value when the value of the quantity is expressed as a number times a unit. In general, the value of a well-defined quantity *A* is expressed as

(1) $$A={\left\{A\right\}}_{\left[A\right]}[A],$$

where {*A*}_[_*_A_*_]_ is the *numerical value* of *A* when the value of *A* is expressed in the unit [*A*]. Equation (1) can be rearranged to read

(2) $$[A]=\frac{A}{{\left\{A\right\}}_{\left[A\right]}},$$

which means that if *A* can be *assumed* to be an invariant quantity, then the unit [*A*] can be *defined* as the quotient of the invariant quantity *A* and a fixed (i.e., exact) numerical value. Simply put, *by fixing the numerical value of an invariant quantity such as the property of an atom or a fundamental constant, we define its unit*.

To illustrate, let us assume that the height of the Washington Monument *h*_WM_ (an obelisk about 169 m tall on the National Mall in Washington D.C. built to commemorate George Washington) is an invariant quantity and that we wish to define a new unit of length for measuring the height of tall buildings called the “washington,” symbol Wa. We could do this by fixing the numerical value of *h*_WM_ to be, for example, exactly 10 when the value of *h*_WM_ is expressed in the unit Wa. It then follows from Eq. (2) that the washington is defined by Wa = *h*_WM_/10, and from Eq. (1) *h*_WM_ = 10 Wa. (Since *h*_WM_ ≈ 169 m, 1 Wa ≈ 16.9 m.)

## 2. Base-Unit Definitions and the Seven Reference Constants of the Current SI

With Sec. 1.2 in mind, we turn our attention to the identification of the set of reference constants on which the current SI is based, by which we mean the set of seven constants (broadly interpreted) whose numerical values are fixed by the seven current SI base-unit definitions. In terms of the discussion in Sec. 1.2, these constants correspond to the quantity *A* on the left-hand-side of Eq. (1) and in the numerator of the right-hand-side of Eq. (2). Our procedure is to first give the current SI definition of the base unit in bold type, and then immediately following the definition the derivation of the associated reference constant. The definitions of the base units are taken from Refs. [1, 2], but for simplicity we do not include any accompanying statements meant to clarify a definition or to indicate how the definition should be realized in practice.

### 2.1 Second and Δ*ν* (^133^Cs)_hfs_

**The second is the duration of 9 192 631 770 periods of the radiation corresponding to the transition between the two hyperfine levels of the ground state of the cesium 133 atom.**

Since the period *T* of a sinusoidal electromagnetic wave is related to its frequency *ν* by the equation *T* = 1/*ν*, if the symbol for the ground state hyperfine splitting transition frequency of the cesium 133 atom is taken to be Δ*ν* (^133^Cs)_hfs_, then it follows from the definition of the second that

(3a) $$s=9192631770\left(\frac{1}{\Delta v{{(}^{133}\text{Cs})}_{\text{hfs}}}\right),$$

or since for a periodic signal s^−1^ = Hz,

(3b) $$\text{Hz}=\frac{\Delta v{{(}^{133}\text{Cs})}_{\text{hfs}}}{9192631770}$$

and thus

(3c) $$\Delta v{{(}^{133}\text{Cs})}_{\text{hfs}}=9192631770\text{Hz}.$$

We see that Eq. (3a) defines s, Eq. (3b) is identical in form to Eq. (2) and defines Hz, and from Eq. (3c), which is identical in form to Eq. (1), that the reference constant whose numerical value is fixed when it is expressed in its SI unit Hz is Δ*ν* (^133^Cs)_hfs_.

### 2.2 Meter and *c*

**The meter is the length of the path travelled by light in vacuum during a time interval of 1/299 792 458 of a second.**

Since *c* is the symbol for the speed of light in vacuum, the length of path *l* travelled by light in vacuum in a time *t* is given by *l* = *ct*. It therefore follows from the definition of the meter that

(4a) $$m=c\left(\frac{1s}{299792458}\right),$$

or

(4b) $${\text{ms}}^{-1}=\frac{c}{299792458}$$

and thus

(4c) $$c=299792458{\text{ms}}^{-1}.$$

In this case we see that although Eq. (4a) formally defines m, it also depends on the definition of s, that Eq. (4b) defines the unit m s^−1^, and from Eq. (4c) that the reference constant whose numerical value is fixed when it is expressed in its SI unit m s^−1^ is *c*. It is important to recognize that the formal definition of the meter does not actually define m but the unit m s^−1^, and thus without a definition of the second the meter is not completely defined by its formal definition.

### 2.3 Kilogram and *m*( K)

**The kilogram is the unit of mass; it is equal to the mass of the international prototype of the kilogram.**

The international prototype of the kilogram (IPK) is denoted by the symbol K, hence the symbol for its mass is simply *m*( K). It therefore follows from the definition of the kilogram that

(5a) kg=m(K)

or

(5b) $$\text{kg}=\frac{m(K)}{1}$$

and thus

(5c) m(K)=1kg.

Here we see that Eq. (5a) formally defines kg, that Eq. (5b) also defines the unit kg, and from Eq. (5c) that the reference constant whose numerical value is fixed when it is expressed in its SI unit kg is *m*( K). Of course, the mass of the IPK is not an invariant of nature and hence not a constant in the sense of Δ*ν* (^133^Cs)_hfs_ and *c*. Indeed, one of the principal motivations for the New SI is the fact that the kilogram is the only SI base unit whose definition is still based on a material artifact.

### 2.4 Ampere and *μ*_0_

**The ampere is that constant current which, if maintained in two straight parallel conductors of infinite length, of negligible circular cross-section, and placed 1 meter apart in vacuum, would produce between these conductors a force equal to 2** × **10^−7^ newton per meter of length.**

The expression from electromagnetic theory for the force *F* per length *l* between two straight parallel conductors a distance *d* apart in vacuum, of infinite length and negligible cross section, and carrying currents *I*_1_ and *I*_2_ is

$$\frac{F}{l}=\frac{\mu_{0}I_{1}I_{2}}{2\pi d},$$

where *μ*_0_ is the magnetic constant (also called “permeability of vacuum”). Applying this expression to the definition of the ampere, we have *F* = 2 × 10^−7^ N, *l* = 1 m, *I*_1_ = *I*_2_ = 1 A, and *d* = 1 m, which leads to

(6a) $$A={\left(\frac{4\pi \times 10^{-7}}{\mu_{0}}N\right)}^{1/2},$$

or

(6b) $${\text{NA}}^{-2}=\frac{\mu_{0}}{4\pi \times 10^{-7}}$$

and thus

(6c) $$\mu_{0}=4\pi \times 10^{-7}{\text{NA}}^{-2}.$$

In this case we see that although Eq. (6a) formally defines A, it also depends on the definitions of s, m, and kg since N = s^−2^ m kg, that Eq. (6b) defines the unit N A^−2^ = s^−2^ m kg A^−2^, and from Eq. (6c) that the reference constant whose numerical value is fixed when it is expressed in its SI unit N A^−2^ = s^−2^ m kg A^−2^ is *μ*_0_. (The value of *μ*_0_ is often expressed in the equivalent unit henry per meter, H m^−1^, where H = s^−2^ m^2^ kg A^−2^.) As in the case of the meter, it is important to recognize that the formal definition of the ampere does not actually define A but the unit N A^−2^ = s^−2^ m kg A^−2^, and thus without definitions of the second, meter, and kilogram the ampere is not completely defined by its formal definition.

### 2.5 Kelvin and *T*_TPW_

**The kelvin, unit of thermodynamic temperature, is the fraction 1 / 273.16 of the thermodynamic temperature of the triple point of water.**

If *T*_TPW_ is taken as the symbol for the thermodynamic temperature of the triple point of water, in principle an invariant quantity, it follows directly from the definition of the kelvin that

(7a) $$K=\frac{T_{\text{TPW}}}{273.16},$$

and hence

(7b) $$T_{\text{TPW}}=273.16K.$$

Here we see that Eq. (7a) formally defines K, and from Eq. (7b) that the reference constant whose numerical value is fixed when it is expressed in its SI unit K is *T*_TPW_.

### 2.6 Mole and *M*(^12^C)

1. **The mole is the amount of substance of a system which contains as many elementary entities as there are atoms in 0.012 kilogram of carbon 12; its symbol is “mol.”**
2. **When the mole is used, the elementary entities must be specified and may be atoms, molecules, ions, electrons, other particles, or specified groups of such particles.**

The molar mass of an entity *X*, symbol *M*(*X*), is the mass of *X* divided by the amount of substance *n* of *X*; its coherent SI derived unit is kilogram per mol, kg mol^−1^. If the entity *X* is the carbon 12 atom, symbol ^12^C, then it follows from the definition of the mole that the molar mass of carbon 12 is 0.012 kg divided by one mole, *M*(^12^C) = 0.012 kg/(1 mol), hence

(8a) $$\text{mol}=\frac{0.012\;\text{kg}}{M{(}^{12}C)},$$

or

(8b) $$\text{kg}\;{\text{mol}}^{-1}=\frac{M{(}^{12}C)}{0.012}$$

and thus

(8c) $$M{(}^{12}C)=0.012\;\text{kg}\;{\text{mol}}^{-1}.$$

In this case we see that although Eq. (8a) formally defines mol, it also depends on the definition of kg, that Eq. (8b) defines the unit kg mol^−1^, and that from Eq. (8c) the reference constant whose numerical value is fixed when it is expressed in its SI unit kg mol^−1^ is *M*(^12^C). Again, as in the case of the meter, it is important to recognize that the formal definition of the mole does not actually define mol but the unit kg mol^−1^, and thus without a definition of the kilogram the mole is not completely defined by its formal definition.

### 2.7 Candela and *K*_cd_

**The candela is the luminous intensity, in a given direction, of a source that emits monochromatic radiation of frequency 540** × **10^12^ hertz and that has a radiant intensity in that direction of 1/683 watt per steradian.**

The luminous intensity of monochromatic radiation in a given direction, *I*_v_, is related to the radiant intensity of the radiation, *I*_e_, and the spectral luminous efficacy of the radiation at the frequency of the radiation, *K*, by the equation *I*_v_ = *I*_e_*K*. Applying this relation to the definition of the candela with *I*_v_ = 1 cd and *I*_e_ = (1/683) W sr^−1^, and denoting *K* at the frequency 540 × 10^12^ Hz by the name luminous efficacy with quantity symbol *K*_cd_, yields

(9a) $$\text{cd}=\frac{K_{\text{cd}}}{683}W\;{\text{sr}}^{-1},$$

or since lm = cd sr,

(9b) $$\text{lm}\;W^{-1}=\frac{K_{\text{cd}}}{683}$$

and thus

(9c) $$K_{\text{cd}}=683\;\text{lm}\;W^{-1}.$$

Here we see that although Eq. (9a) formally defines cd, it also depends on the definitions of s, m, and kg since W = s^−3^ m^2^ kg and sr = m^2^ m^−2^ = 1, that Eq. (9b) defines the unit lm W^−1^ = s^3^ m^−2^ kg^−1^ cd sr since lm = cd sr, and from Eq. (9c) that the reference constant whose numerical value is fixed when it is expressed in its SI unit lm W^−1^ = s^3^ m^−2^ kg^−1^ cd sr is *K*_cd_. Once again it is important to recognize that the formal definition of the candela does not actually define cd but the unit lm W^−1^ = s^3^ m^−2^ kg^−1^ cd sr, and thus without definitions of the second, meter, and kilogram, the candela is not completely defined by its formal definition.

### 2.8. Explicit-Unit vs. Explicit-Constant Definitions

The definitions of the current SI base units in Secs. 2.1–2.7 are now called “explicit unit,” because the exact value of the constant to which the unit is linked is specified indirectly by explicitly defining the unit in terms of a quantity of the same kind as the unit [4, 5]. They should be compared with those for the same units as given in Part II of the definition of the New SI in Sec. 5, Appendix B. These are of a form that explicitly indicates the constant to which the unit is linked. This form is called “explicit-constant,” because the unit is defined indirectly by specifying explicitly an exact numerical value for a constant of nature [4, 5]. The formulation of the definition of the New SI as given in Appendix B, which begins with an overall scaling statement followed by base-unit definitions in explicit-constant form, has the great advantage that the foundation of the New SI is uncovered for all to see. Moreover, the explicit-constant form has the additional advantage that each base unit can be defined in the same way, thereby increasing the uniformity of their definitions and one would expect their understandability.

## 3. Conclusion

In summary, we have shown explicitly in Secs. 2.1–2.7 that in the current SI the ensemble of base units, s, m, kg, A, K, mol, and cd, are actually defined by fixing the numerical values of the set of seven reference constants Δ*ν* (^133^Cs)_hfs_, *c*, *m*( K), *μ*_0_, *T*_TPW_, *M*(^12^C), and *K*_cd_ when these constants are expressed in the units s, m s^−1^, kg, N A^−2^, K, mol^−1^, and lm W^−1^, respectively, and as a result, the values of these seven constants in SI units are exactly known.

Equally as important, we have shown in Sec. 6, Appendix C how the definition of the current SI can be recast into a fully equivalent form that is identical to the two-part form proposed for defining the New SI by the CIPM and which is under consideration by the CGPM for eventual adoption. Thus, putting aside the difference in the wordings of the definitions, the principal metrological difference between the current SI and the New SI is that the group of four reference constants *m*( K), *μ*_0_, *T*_TPW_, and *M*(^12^C) in the current SI is replaced in the New SI by the group *h*, *e*, *k*, and *N*_A_.

Of course, the difference in wordings is highly significant, because the New SI wording makes eminently clear the constants and their values in terms of which it is defined; this is certainly not the case for the wording of the current SI. Indeed, as discussed in Sec. 7, Appendix D, the Part I simple but clear form of definition of the New SI can be viewed as a more general, easier to understand, and transparent way to formulate a system of units.

As indicated in Sec. 4, Appendix A, the CGPM formally established the SI in 1960, some 50 years ago. Nevertheless, it was not until the publication by the BIPM of the 7th edition of the SI Brochure in 1998 that the effect of the base-unit definitions on the values of certain constants was begun to be widely noted. In particular, in this edition the formal definitions of the meter and ampere were followed by the statements “Note that the effect of this definition is to fix the speed of light at exactly 299 292 458 m s^−1^,” and “Note that the effect of this definition is to fix the permeability of vacuum at exactly 4π × 10^−7^ H m^−1^,” respectively. It was not until the 8th edition of the SI brochure published in 2006 [1] that a similar explanatory statement followed each formal base-unit definition. Moreover, to the best of the author’s knowledge, there has never been a published paper prior to this one that explicitly shows how these definitions lead to exact values of Δ*ν* (^133^Cs)_hfs_, *c*, *m*( K), *μ*_0_, *T*_TPW_, *M*(^12^C), and *K*_cd_.

One may well wonder why this useful way of viewing the SI did not gain prominence sooner. Although we have no real answer to this question, a motivating factor for the inclusion of the two explanatory statements in the 7th edition of the SI Brochure was likely the discussion by Ulrich Feller of the national metrology institute of Switzerland at the 21st meeting of the CIPM’s Consultative Committee on Electricity (CCE) of document CCE/97-3, which he had submitted to the meeting [13, 14]. In it, Feller points out the dependence of five of the SI base units on fundamental constants.

## Figures and Tables

**Table 1:** Concise summary of the current SI base quantities and units (adapted from Refs. [1, 2] and unchanged in the New SI)

Base quantity	Base unit	
Name	Name	Symbol
time	second	s
length	meter	m
mass	kilogram	kg
electric current	ampere	A
thermodynamic temperature	kelvin	K
amount of substance	mole	mol
luminous intensity	candela	cd

**Table 2:** Concise summary of the coherent derived units in the current SI with special names and symbols that appear in this paper (adapted from Refs. [1, 2] and unchanged in the New SI)

Derived quantity	SI coherent derived unit		Expressed in terms of other SI units	Expressed in terms of SI base units
	Name	Symbol		
solid angle	steradian	sr		m^2^ m^−2^ = 1
frequency	hertz	Hz		s^−1^
force	newton	N		s^−2^ m kg
energy	joule	J	Nm	S^−2^ m^2^ kg
power	watt	W	Js^−1^	s^−3^ m^2^ kg
electric charge	coulomb	C		s A
voltage	volt	V	WA^−1^	s^−3^ m^2^ kg A^−1^
electric resistance	ohm	Ω	VA^−1^	s^−3^ m^2^ kg A^−2^
inductance	henry	H	V s A^−1^	s^−2^ m^2^ kg A^−2^
luminous flux	lumen	lm	cd sr	m^2^ m^−2^ cd = cd

**Table 3:** The New SI as defined in Part I of Sec. 5, Appendix B viewed as an alternative formulation of a system of units. The words “per” and “by” are to be interpreted to mean “divided by” and “times,” respectively. Column one gives the set of seven reference constants of the New SI, columns two and three the kind of quantity and its commonly used symbol of which each reference constant is a particular example, and columns four and five the unit and its symbol defined by assigning an exact numerical value to the corresponding reference constant. The last two columns show how the kind of quantity of which each reference constant is a particular example can be expressed in terms of other quantities of the system of quantities. (*N* is the quantity symbol for cycles, which is a quantity of dimension one and can be a decimal number; its unit is the number one [1, 2]. *t*, *l*, *I*, and *m* are quantity symbols for time, length (distance), electric current, and mass, respectively)

Reference constant	Kind of quantity and its symbol		Unit and its symbol fixed by reference constant		Quantity in terms of other quantities and their symbols	
Δ*ν* (^133^Cs)_hfs_	frequency	*ν*	hertz	Hz	cycles per time	*N*/*t*
*c*	velocity	*υ*	meter per second	m s^−1^	length per time	*l*/*t*
*h*	action	*S*	joule second	J s	energy by time	*Et*
*e*	charge	*Q*	coulomb	C	current by time	*It*
*k*	(change in) energy per (change in) thermodynamic temperature	*E*/*T*	joule per kelvin	J K^−1^	mass by velocity squared per thermodynamic temperature	*mυ*^2^/*T*
*N*_A_	reciprocal amount of substance	*n*^−1^	reciprocal mole	mol^−1^		
*K*_cd_	luminous flux per power	*Φ*_v_/*P*	candela steradian per watt	cd sr W^−1^	luminous flux per energy per time	*Φ*_v_/(*E*/*t*)

## 4. Appendix A. Background and Brief Overview of the Current SI

The International System of Units (SI) was formally established by the 11th General Conference on Weights and Measures (CGPM) in 1960 and has been modified a number of times by the CGPM over the last 50 years to reflect advances in both science and technology [1, 2]. The CGPM, a diplomatic organization consisting of delegates from the Member States of the Meter Convention signed in Paris in 1875 by 17 countries, including the United States, and which usually meets every 4 years, is one of the three principal organs created by the Convention (at present 55 countries are signatories). The two others are the International Committee for Weights and Measures (CIPM) and the International Bureau of Weights and Measures (BIPM). The CIPM has 18 members, each from a different Member State, and at present meets yearly. The BIPM, located in Sèvres, France, just outside of Paris, and whose task is to ensure world-wide uniformity of measurements and their traceability to the SI, operates under the supervision of the CIPM, which itself is under the authority of the CGPM.

The CIPM has established 10 Consultative Committees (CCs) which together cover all fields of basic metrology; for example, there is the Consultative Committee for Electricity and Magnetism (CCEM) and the Consultative Committee for Length (CCL) [1, 2]. The purpose of the CCs is to provide the CIPM information on matters that it refers to them for study and advice. A CC of particular relevance to the subject of this paper is the Consultative Committee on Units (CCU), in whose work the author has been an active participant for the past 20 years.

One of the CCU’s most important tasks is to prepare, under the guidance of the CIPM, an official and definitive description of the SI. Published by the BIPM and commonly called the SI Brochure, the 1st edition of the Brochure appeared in 1970 and the 8th Edition, the most recent, in 2006; it may be downloaded free of charge from the BIPM’s Web site [1]. A version of the SI Brochure prepared and published by NIST specifically for use in the United States is also available and may be downloaded free of charge from NIST’s Web site [2].

The current SI is founded on the seven base quantities, assumed to be independent, time, length, mass, electric current, thermodynamic temperature, amount of substance, and luminous intensity, for which the SI base units are the second s, meter m, kilogram kg, ampere A, kelvin K, mole mol, and candela cd, respectively; see Table 1.

For convenience, “coherent derived units” (see following paragraph) that are particular combinations of base units, or in two cases a different form of a particular base unit, are given special names and symbols. An example is the joule J, the coherent derived SI unit for the derived quantity energy: “joule” is the special name for the “kilogram meter squared per second squared” and “J” is the special symbol for “s^−2^ m^2^ kg.” (Throughout this paper, when derived units with special symbols are expressed in terms of their component base-unit symbols, the exponential form of the latter is used and the order s, m, kg, A, K, mol, and cd is followed.) For easy reference, the special units that appear in this paper are summarized in Table 2 (all 22 can be found in Table 3 of Refs. [1, 2]).

As explained in Sec. 1.4 of Refs. [1, 2], derived units are products of powers of base units. When the product includes no numerical factor other than the number one, the derived units are *coherent derived units*. The SI base units and SI coherent derived units, which include the 22 derived units with special names and symbols, form a coherent set. Such units have the great advantage that the equations between the numerical values of quantities (see Sec. 1.2) are of the exact same form as the equations between the quantities themselves, and hence conversion factors between units are never required.

Finally, there are the SI prefixes for forming multiples and submultiples of coherent units in order to express values of quantities significantly larger or smaller than the units. The 10 SI prefixes for multiples cover the range 10^1^ (deka, da) to 10^24^ (yotta, Y), and include the familiar kilo, k (10^3^) and mega, M (10^6^); the 10 SI prefixes for submultiples cover the range 10^−1^ (deci, d) to 10^−24^ (yocto, y), and include the familiar milli, m (10^−3^) and micro, μ (10^−6^). All 20 SI prefixes can be found in Table 5 of Refs. [1,2].

## 5. Appendix B. Proposed New SI

The New SI as presently envisioned is presented in Refs. [10, 11]. In brief, its definition contains two principal parts: Part I is a “scaling statement” [5] that defines the new system by fixing the magnitudes of an ensemble of units by assigning exact numerical values to a set of seven reference constants expressed in those units without reference to base and derived units; Part II contains the individual definitions of the base units s, m, kg, A, K, mol, and cd using the same set of reference constants. These are the ground state hyperfine splitting frequency of the cesium 133 atom Δ*ν* (^133^Cs)_hfs_; the speed of light in vacuum *c*; the Planck constant *h*; the elementary charge *e* (charge of a proton); the Boltzmann constant *k*; the Avogadro constant *N*_A_, and the luminous efficacy *K*_cd_ of monochromatic radiation of frequency 540 × 10^12^ hertz. Although the scaling statement is itself sufficient to define the entire system, in recognition of the historical structure of the SI and the utility of the concepts of base and derived units, the New SI retains these concepts. In particular, it retains the seven base units as given in Table 1 and all 22 of the derived units with special names and symbols, some of which are given in Table 2.

The version of the New SI presented here is adapted from Ref. [10], but some comparatively minor word changes before eventual adoption are possible. The numbers in the definitions are based on the 2010 Committee on Data for Science and Technology (CODATA) set of recommended values of the constants first available starting 2 June 2011 at http://physics.nist.gov/constants. The ellipses indicate additional digits to be added when the New SI is adopted and are expected to be provided by CODATA.

**Part I**

**The International System of Units, the SI, is the system of units in which:**

- the ground state hyperfine splitting frequency of the cesium 133 atom Δ*ν* (^133^Cs)_hfs_ is exactly 9 192 631 770 hertz,
- the speed of light in vacuum *c* is exactly 299 792 458 meter per second,
- the Planck constant *h* is exactly 6.626 069…× 10^−34^ joule second,
- the elementary charge *e* is exactly 1.602 176…× 10^−19^ coulomb,
- the Boltzmann constant *k* is exactly 1.380 648…× 10^−23^ joule per kelvin,
- the Avogadro constant *N*_A_ is exactly 6.022 141…× 10^23^ reciprocal mole,
- the luminous efficacy *K*_cd_ of monochromatic radiation of frequency 540 × 10^12^ Hz is exactly 683 lumen per watt.

**Part II**

**The second, symbol s, is the unit of time; its magnitude is set by fixing the numerical value of the ground state hyperfine splitting frequency of the cesium 133 atom to be equal to exactly 9 192 631 770 when it is expressed in the SI unit s^−1^, which is equal to Hz.**

**The meter, symbol m, is the unit of length; its magnitude is set by fixing the numerical value of the speed of light in vacuum to be equal to exactly 299 792 458 when it is expressed in the SI unit m s^−1^.**

**The kilogram, symbol kg, is the unit of mass; its magnitude is set by fixing the numerical value of the Planck constant to be equal to exactly 6.626 069…** × **10^−34^ when it is expressed in the SI unit s^−1^ m^2^ kg, which is equal to J s.**

**The ampere, symbol A, is the unit of electric current; its magnitude is set by fixing the numerical value of the elementary charge to be equal to exactly 1.602 176…** × **10^−19^ when it is expressed in the SI unit s A, which is equal to C.**

**The kelvin, symbol K, is the unit of thermodynamic temperature; its magnitude is set by fixing the numerical value of the Boltzmann constant to be equal to exactly 1.380 648…** × **10^−23^ when it is expressed in the SI unit s^−2^ m^2^ kg K^−1^, which is equal to J K^−1^.**

**The mole, symbol mol, is the unit of amount of substance of a specified elementary entity, which may be an atom, molecule, ion, electron, any other particle or a specified group of such particles; its magnitude is set by fixing the numerical value of the Avogadro constant to be equal to exactly 6.022 141…** × **10^23^ when it is expressed in the SI unit mol^−1^.**

**The candela, symbol cd, is the unit of luminous intensity in a given direction; its magnitude is set by fixing the numerical value of the luminous efficacy of monochromatic radiation of frequency 540** × **10^12^ Hz to be equal to exactly 683 when it is expressed in the SI unit s^3^ m^−2^ kg^−1^ cd sr, or cd sr W^−1^, which is equal to lm W^−1^.**

## 6. Appendix C. Recasting the Definition of the Current SI

Recasting the definition of the current SI into the form of the New SI is straightforward, because essentially all that is required is to replace the four reference constants *h*, *e*, *k*, and *N*_A_ of the New SI with the four reference constants *m*(*K*), *μ*_0_, *T*_TPW_, and *M*(^12^C) of the current SI. If written in the form of the New SI, the current SI might look something like the following:

Part I

The International System of Units, the SI, is the system of units in which:

- the ground state hyperfine splitting frequency of the cesium 133 atom Δ*ν* (^133^Cs)_hfs_ is exactly 9 192 631 770 hertz,
- the speed of light in vacuum *c* is exactly 299 792 458 meter per second,
- the mass of the international prototype of the kilogram *m*( K) is exactly 1 kilogram,
- the magnetic constant *μ*_0_ is exactly 4π × 10^−7^ newton per ampere squared,
- the triple point of water *T*_TPW_ is exactly 273.16 kelvin,
- the molar mass of carbon 12 *M*(^12^C) is exactly 0.012 kilogram per mole,
- the luminous efficacy *K*_cd_ of monochromatic radiation of frequency 540 × 10^12^ hertz is exactly 683 lumen per watt.

Part II

The second, symbol s, is the unit of time; its magnitude is set by fixing the numerical value of the ground state hyperfine splitting frequency of the cesium 133 atom to be equal to exactly 9 192 631 770 when it is expressed in the SI unit s^−1^, which is equal to Hz.

The meter, symbol m, is the unit of length; its magnitude is set by fixing the numerical value of the speed of light in vacuum to be equal to exactly 299 792 458 when it is expressed in the SI unit m s^−1^.

The kilogram, symbol kg, is the unit of mass; its magnitude is set by fixing the numerical value of the mass of the international prototype of the kilogram to be equal to exactly 1 when it is expressed in the SI unit kg.

The ampere, symbol A, is the unit of electric current; its magnitude is set by fixing the numerical value of the magnetic constant to be equal to exactly 4π × 10^−7^ when it is expressed in the SI unit s^−2^ m kg A^−2^, which is equal to N A^−2^.

The kelvin, symbol K, is the unit of thermodynamic temperature; its magnitude is set by fixing the numerical value of the triple point of water to be equal to exactly 273.16 when it is expressed in the SI unit K.

The mole, symbol mol, is the unit of amount of substance of a specified elementary entity, which may be an atom, molecule, ion, electron, any other particle or a specified group of such particles; its magnitude is set by fixing the numerical value of the molar mass of carbon 12 to be equal to exactly 0.012 when it is expressed in the SI unit kg mol^−1^.

The candela, symbol cd, is the unit of luminous intensity in a given direction; its magnitude is set by fixing the numerical value of the luminous efficacy of monochromatic radiation of frequency 540 × 10^12^ Hz to be equal to exactly 683 when it is expressed in the SI unit s^3^ m^−2^ kg^−1^ cd sr, or cd sr W^−1^, which is equal to lm W^−1^.

## 7. Appendix D. Alternative Formulation of a System of Units

Probably the most familiar approach to the formulation of a system of units such as the SI is the following [1, 2]: A system of quantities is established, which includes a set of equations that define the relations among the quantities. The system of quantities comprises all of the familiar quantities of physics such as mass *m*, acceleration *a*, and force *F* and the equations among them such as *F* = *ma*. A small number of *base quantities* that are assumed to be independent are chosen from the system of quantities such that any other quantity of the system, called a *derived quantity*, can be expressed as a function of the base quantities. Units for the base quantities, called *base units*, are chosen and defined, and *derived units* for the derived quantities are obtained as products of the base units using the same functions.

An alternative approach, first proposed in Ref. [5] and due to P. J. Mohr, and which recognizes from the start that the end result of the more familiar approach is a set of reference constants with exactly known values that defines the system of units, is exemplified by Part I of the New SI given in Sec. 5, Appendix B. The starting point is the same system of quantities, which in the case of interest here is that on which the current SI is based. But the next step, which takes full advantage of the laws of physics and our ability to determine experimentally the values of certain constants of nature with very small uncertainties, is the selection of a set of such constants that depend on quantities of the system of quantities and that together can be used as a set of reference constants for defining a practical system of units. As discussed in Sec. 1.2, this system of units is established by assigning an exact numerical value to each reference constant in the set, thereby defining its unit.

Of course, the chosen set of reference constants must be complete and non-redundant. By complete is meant that the units defined by fixing the numerical value of each reference constant in the set must be sufficient to enable the expression of the measured value of any particular kind of quantity in the system of quantities; by non-redundant is meant that no unit defined by the fixed numerical value of one reference constant in the set is independently defined by a combination of the other constants in the set; see the paper by Mohr [6]. In this formulation all units are viewed as being on an equal footing—there are no “base units” or “derived units.” Table 3 shows in detail how Part I of the New SI given in Sec. 6, Appendix B exemplifies this alternative formulation.

Factors to be considered when choosing a set of reference constants on which to base a system of units (see Ref. [11] for additional details) include (i) the extent to which the choice reduces the uncertainties of practical measurements; (ii) the ease of realizing with the requisite uncertainty the units defined by the reference constants; and (iii) the impact of the choice on our knowledge of the values of the fundamental constants and on the periodic adjustment of their values by CODATA. The decision to replace *m*( K), *μ*_0_, *T*_TPW_, and *M*(^12^C) in the current SI with *h*, *e*, *k*, and *N*_A_ to establish the New SI is based in part on experimental advances made over the last several decades in using the Josephson effect (JE) to measure voltage, the quantum hall effect (QHE) to measure resistance, and acoustic gas thermometry (AGT) to measure thermo-dynamic temperature [11]. Further, with *h* and *N*_A_ exactly known, recent improvements in our knowledge of the values of the Rydberg constant, fine-structure constant, and relative atomic mass (formerly called “atomic weight”) of the electron allow the molar mass of an entity *X* to be obtained with negligible increase in uncertainty from its own relative atomic mass, thereby facilitating the determination of the amount of substance of a sample of *X* [11].

It is beyond the scope of this appendix to explore any further the alternative formulation of a system of units as outlined here, but we conclude with the following observations:

- The completeness of the New SI is assured because it defines the units s, m, kg, A, K, mol, and cd, which are the base units of the current SI. Indeed, any set of reference constants that does this is complete.
- The four New SI reference constants *h*, *e*, *k*, and *N*_A_ should not be viewed as being unique in the sense that they are the only ones that could have been chosen. For example, the Josephson and von Klitzing constants *K*_J_ and *R*_K_ could have been selected in place of *h* and *e*. Since *K*_J_ is an example of the kind of quantity frequency per voltage, and *R*_K_ is an example of the kind of quantity electric resistance, the units that are defined by assigning exact numerical values to these constants are hertz per volt, Hz/V, and ohm, Ω, respectively.
- Abandoning the distinction between base units and derived units does not eliminate the concept “dimension of a quantity” nor preclude dimensional analysis [1, 2]. Such analysis could be carried out exactly as at present or in terms of any complete set of quantities and their units. One such set is time, length, energy, charge, thermodynamic temperature, amount of substance, and luminous intensity with units s, m, J, C, K, mol, and cd.
